# Natural grasping movement recognition and force estimation using electromyography

**DOI:** 10.3389/fnins.2022.1020086

**Published:** 2022-10-20

**Authors:** Baoguo Xu, Kun Zhang, Xinhao Yang, Deping Liu, Cong Hu, Huijun Li, Aiguo Song

**Affiliations:** ^1^The State Key Laboratory of Bioelectronics, Jiangsu Key Laboratory of Remote Measurement and Control, School of Instrument Science and Engineering, Southeast University, Nanjing, China; ^2^Guangxi Key Laboratory of Automatic Detecting Technology and Instruments, Guilin University of Electronic Technology, Guilin, China

**Keywords:** natural grasping movements, grasping force, electromyography (EMG), action decoding, force estimation

## Abstract

Electromyography (EMG) generated by human hand movements is usually used to decode different action types with high accuracy. However, the classifications of the gestures rarely consider the impact of force, and the estimation of the grasp force when performing natural grasping movements is so far overlooked. Decoding natural grasping movements and estimating the force generated by the associated movements can help patients to improve the accuracy of prosthesis control. This study mainly focused on two aspects: the classification of four natural grasping movements and the force estimation of these actions. For this purpose, we designed an experimental platform where subjects could perform four common natural grasping movements in daily life, including pinch, palmar, twist, and plug grasp, to complete target profiles. On the one hand, the results showed that, for natural grasping movements with different levels of force (three levels at 20, 50, and 80%), the average accuracy could reach from 91.43 to 97.33% under five classification schemes. On the other hand, the feasibility of force estimation for natural grasping movements was demonstrated. Furthermore, in the process of force estimation, we confirmed that the regression performance about plug grasp was the best, and the average *R*^2^ could reach 0.9082. Besides, we found that the regression results were affected by the speed of force application. These findings contribute to the natural control of myoelectric prosthesis and the EMG-based rehabilitation training system, improving the user’s experience and acceptance.

## Introduction

Electromyography (EMG) is a typical bioelectrical phenomenon, which is the measurement of muscle electrical activity during voluntary contraction or after nerve stimulation. Surface EMG (sEMG) is the EMG signal measured on the skin surface by a non-invasive technique. Many EMG signals are generated by the muscles of the arm along with the execution of various hand movements (Scheme and Englehart, 2011). In this process, EMG contains much information about motions and forces. Studies have shown that the terminal muscle nerves of amputees are still active (Tan et al., 2015; Cracchiolo et al., 2020) and the extraction of biological signals from arm muscles can help disabled people with motion assistance and prosthetic control (Nielsen et al., 2011; Nizamis et al., 2020). Taborri et al. (2018) gave a review on the present study of the muscle synergy theory, which indicated that it is possible and promising to apply this theory to the fields of clinics, sports, and robotics. Castellini et al. devised an experiment specifically for below-elbow amputees for the first time. They found that the accuracy of the posture classification ranged from 79.72 to 95.74% and that the RMSE of the estimated force ranged from 6.54 to 17.76% after being normalized. The results demonstrated that sEMG could be utilized to help the amputees (Castellini et al., 2009). Therefore, studying the classification and force estimation of natural grasping movements based on EMG is of great significance for the daily life of the disabled (Peerdeman et al., 2011; Farina et al., 2014; Qi et al., 2019).

In the past decades, gesture recognition has made great achievements. Zhang et al. proposed a framework for hand gesture recognition based on the fusion of a three-axis accelerometer and multichannel EMG sensor. This framework got an accuracy of more than 95% in recognizing 72 Chinese Sign Language words through the combination of decision tree and multistream hidden Markov models (Zhang et al., 2011). In the study of Castellini et al., the principal component analysis (PCA) was used to extract the effective information of sEMG signals and the general regression neural network (GRNN) was applied to recognize nine static gestures. The classification accuracy could reach 95.1% (Castellini et al., 2008). Geng et al. (2016) introduced a new concept of sEMG image, which could be utilized to recognize numerous gestures only with sEMG signals at a specific instant. Shenoy et al. (2008) obtained 92–98% accuracy by linear support vector machines for offline processing of eight gestures. The bilinear model proposed by Matsubara et al. successfully recognized five gestures and controlled a robotic arm (Matsubara and Morimoto, 2013). Khushaba et al. (2021) proposed a new method called Recurrent Spatial-Temporal Fusion, which significantly outperformed all other traditional pattern recognition methods or deep learning approaches. Blana et al. (2020) applied a biomechanical model to prosthesis control, and the average correlation between the model and real movement reached 0.89. Sierotowicz et al. (2022) proposed a soft glove that can detect the user’s motion intention by EMG to help patients with grip assistance and rehabilitation training. Xie et al. (2021) also made an exoskeleton for hand movement to help stroke patients reach and grasp. However, to the best of our knowledge, although studies focusing on the recognition of gestures based on sEMG have been widely conducted, there is still a lack of the decoding of natural grasping movements, such as pinch, palmar, twist, plug grasp, etc. Furthermore, most classification schemes didn’t consider the force of natural movements and it is worth exploring the impact of force in classifying natural grasping movements.

Meanwhile, compared to the classification of gestures by EMG, little research has been done on the force estimation of natural grasping movements. Martinez et al. proposed to predict palmar force by high-density EMG, which reached an absolute error of 2.52% offline. Then, they got an absolute error of 2.06% (for non-amputees) and 2.04% (for amputees) online (Martinez et al., 2020a,b). Zhang et al. (2018) applied a fast independent component analysis to decompose the high-density EMG, and then used K-means clustering to extract the input signal for force estimation, with *R*^2^ reaching 0.877–0.955. Moreover, some researchers set different force levels and target profiles to observe the effect of EMG in experiments (Boudaoud et al., 2015; Li et al., 2018). Hoozemans and van Dieen (2005) also found that the regression performance of three muscles was not worse than six muscles when using forearm muscles to estimate hand grip force, which means that we need to choose different muscle channels to avoid mutual interference. Nevertheless, most grasping movements have not been studied yet, such as twist grasp and plug grasp, which are commonly involved in the fields of rehabilitation, especially for natural control of the prosthesis. In addition, the force curve is another aspect that deserves attention. Solomonow et al. (1986) mentioned that the appropriate selection of force curves should take the recruitment pattern of muscles into consideration. Kamavuako et al. (2009) obtained the results that different force profiles may lead to different consequences under certain conditions. However, the force curves in many experiments are set at will without considering the impact of the force curves. And the effects of different parts of muscles on force estimation were not investigated deeply.

In this manuscript, we aimed to investigate the classification and continuous force estimation of four natural grasping movements when different forces were involved. The experimental apparatus was designed to perform these actions: pinch, palmar, twist, and plug grasp. In addition, different classification schemes involving force information were evaluated by EMG. In terms of force estimation, we designed four target profiles to simulate the force application mode in real-life situations.

## Materials and methods

### Subjects

Five able-bodied subjects (aged 22–26, right-handed) participated in this experiment. All subjects had no previous experience with this study and had signed an informed consent form. The experiments were conducted at Southeast University, Nanjing, China. The experimental protocols were approved by the Ethics Committee of Southeast University and did not cause any harm to humans. To avoid bad muscle conditions during the experiment, each subject was asked to be prohibited from participating in strenuous upper limb exercises before the experiment.

### Experimental setup and paradigm

**FIGURE 1:**
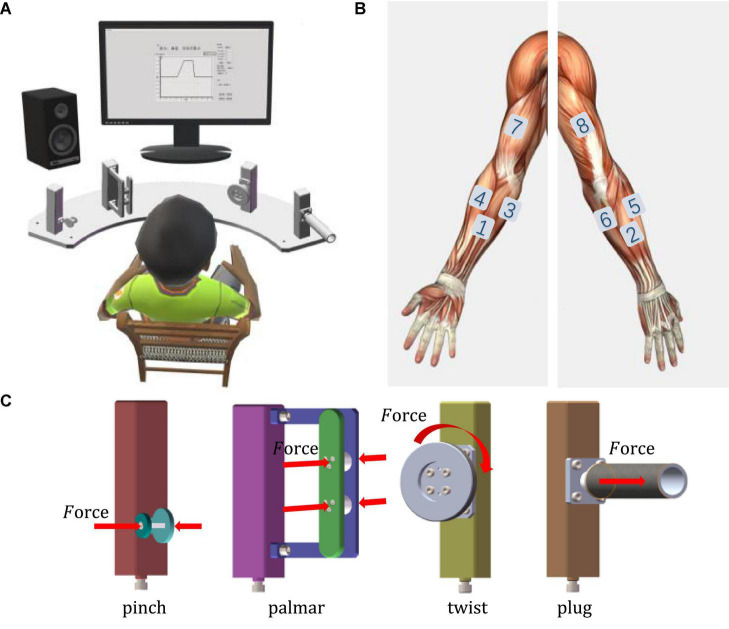
Experimental setup for four natural grasping movements. **(A)** The experimental table with a screen in front. **(B)** EMG electrode setup on right arm (left: front, right: back). **(C)** Force sensor measurement direction.

**FIGURE 2:**
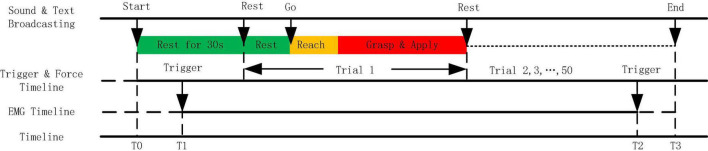
The experimental paradigm based on the audio and visual cues and data synchronization scheme.

During the experiment, the subject sat in a comfortable chair and performed four natural grasping movements, always in a natural and relaxed state (Figure 1A). The fan-shaped platform, which was composed of a pinch device, a grip device, a twist device, and a plug device from left to right, was designed to complete four movements: pinch, palmar grasp, twist, and plug grasp. The forces were measured in the directions as illustrated in Figure 1C. Eight wireless electrodes were placed on the surface of the forearm and the upper limb (Figure 1B). A 24-inch monitor displayed the target force profile and the real-time force application curve to ensure the accuracy of the subject’s hand motion execution. The subject completed the experimental task according to the sound and screen. Figure 2 shows the experimental paradigm based on the audio, visual cues, and data synchronization scheme. Trigger signals were adopted to synchronize EMG signal and force data. When the trigger was pressed, there would be a pulse in the signal. As the experiment started, the upper computer began to sample data and instructed the subject. After the initial 30 s rest, there were 50 trials immediately following. The acquisition of force and trigger signals started at T0 and stopped at T3, and the EMG acquisition started at T1 and stopped at T2. The whole experiments were divided into two parts: classification and force estimation. Before each experiment, the subject was required to perform each natural grasping movement with maximum voluntary contraction (MVC) thrice. The calculated average value was used as the final reference value.

**FIGURE 3:**
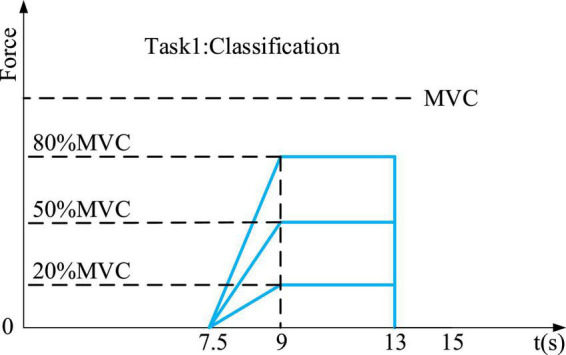
Three profiles to apply force in the first experiment. Blue lines represent the target force profiles.

**FIGURE 4:**
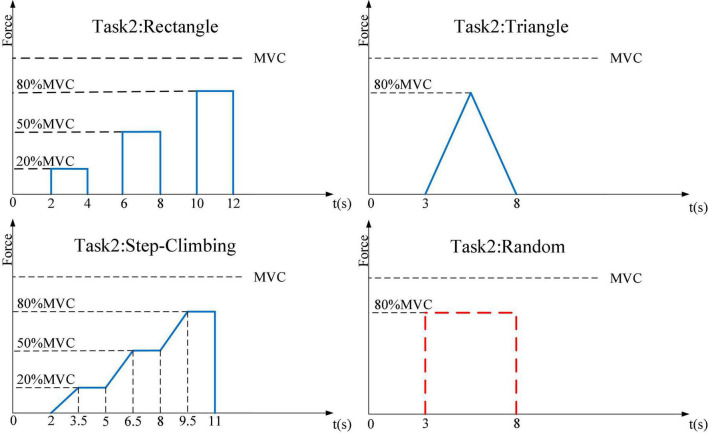
Four profiles to apply force in the second experiment. Blue lines represent the target profile. In the random profile, the red dotted line represents that the subject should not exert force beyond this range.

The first experiment was designed for the classification of the four natural grasping movements with force information and required subjects to: (1) move their right hand to the experimental device and perform the corresponding hand action, (2) apply force in three ways: a. reach 20% MVC in 1.5 s and hold for 4 s; b. reach 50% MVC in 1.5 s and hold for 4 s; c. reach 80% MVC in 1.5 s and hold for 4 s, and (3) move the right hand back to the center to rest and wait for the instruction of the next round (see Figure 3). The second experiment adopted four target profiles for force estimation, which were: (1) a rectangular profile of 20% MVC, 50% MVC, and 80% MVC, each with a width of 2 s, (2) a triangle profile with a maximum of 80% MVC, lasting 5 s, (3) a step-climbing profile of 8.5 s, and (4) a random profile within 5 s, but required to reach 80% MVC at most during force application (see Figure 4).

For the first experiment, subjects were instructed to perform four natural movements under three target forces: 20% MVC, 50% MVC, and 80% MVC, respectively. Each session consisted of 50 trials of 13 s length, including 7 s rest time, 0.5 s reaching time, 1.5 s lifting force, and 4 s holding force. For the second experiment, four target profiles were performed by subjects for hand actions. Each target profile contained 20 trials and one trial lasted 13 s (as shown in Table 1). Furthermore, subjects were asked to familiarize themselves with the grasps and target profiles. In addition, subjects were given a rest period of about 10 min between each session to avoid muscle fatigue (Downey et al., 2017). Each experiment took about 4 h.

**TABLE 1:** Target grasping force for different experiments.

Experiment	Target grasping force	Trials
First experiment	20% MVC	50
	50% MVC	50
	80% MVC	50
Second experiment	A rectangular profile	20
	A triangle profile	20
	A step-climbing profile	20
	A random profile	20

### Signal acquisition

The EMG acquisition system (Delsys Inc., Natick, MA, USA) was adopted in this experiment, and eight wireless channels with a sampling frequency of 2000 Hz were used. Before electrode sticking, subjects were asked to clean the skin surface with alcohol. The positions of the eight electrodes were flexor carpi radialis, extensor digitorum, palmaris longus, brachioradialis, extensor carpi radialis, extensor carpi ulnaris, biceps brachii, and triceps brachii, representing eight channels {C1, C2, C3, C4, C5, C6, C7, C8} (see Figure 1B). EMG signals were filtered by using a sixth-order Butterworth filter of 10–500 Hz and a notch filter at 50 Hz. The force of the grasping movements was recorded by miniature transducers inside the grasp handles. The force data and trigger signal were sent to the host computer by a data acquisition card. The force signal was also sampled at 2000 Hz.

### Data processing

The EMG and force signals were processed offline, and the data were segmented and characterized with the sliding window approach. We selected five time-domain features as input for classification and force estimation, which were Mean Absolute Value (MAV), Root Mean Square (RMS), Variance (VAR), Willison Amplitude (WAMP), and Waveform Length (WL) (Phinyomark et al., 2012, 2013; Wang et al., 2019).

For the first experiment, we classified different natural grasping movements and studied the relationship between movements and muscles. The grasp force phase, which is the 10∼13 s time of the experiment, was selected for data analysis. Moreover, grasping force was divided into three levels (level 1: 20% MVC, level 2: 50% MVC, and level 3: 80% MVC). Five classification schemes (SCH) were proposed to investigate the effect of grasping force on the four natural grasping movements, which were: (1) performing four-class classification for these actions, without distinguishing three levels of force, (2) performing eight-class classification for these actions, with level1 and level 2, (3) performing eight-class classification for these actions, with level 2 and level 3, (4) performing eight-class classification for these actions, with level 1 and level 3, and (5) performing 12-class classification for these actions, with level 1, level 2, and level 3. We selected a sliding window with a window width of 175 ms and a step size of 20 ms to process the data. The Support Vector Machine (SVM) was selected as the classifier and the robustness was evaluated *via* 5-fold cross-validation.

For the second experiment, we selected a Back Propagation (BP) network (Zhang, 2019) as the force regression model. The inputs of the BP network were the eigenvectors that consisted of different features extracted from each channel, such as root mean square, mean absolute value, Willison amplitude, and so on. The outputs were the force regression values corresponding to the different movements. By comparing different window widths and step sizes, a window width of 200 ms and a step size of 80 ms were chosen, and the force was the average of this sliding window. We trained the regression model with the data segments and obtained the non-linear relationship between the EMG features and the force. We also evaluated the relationship between eight muscles and four natural grasping movements to select the best channels for force estimation. In this paper, the regression performance of the model was evaluated by the coefficient of determination, *R*^2^.

## Results

### Classification with force information

**FIGURE 5:**
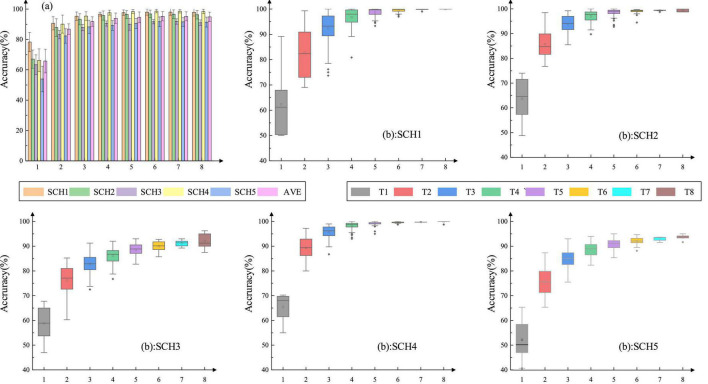
Average recognition rate about the five classification schemes under different total numbers of channels and the box diagram of subject one. The horizontal axis represents the total number of channels. SCH1: four-class classification for four actions without using force information. SCH2: eight-class classification for four actions with level 1 and level 2 of force. SCH3: eight-class classification for four actions with level 2 and level 3 of force. SCH4: eight-class classification for four actions with level1 and level3 of force. SCH5: 12-class classification for four actions with level1, level2, and level3 of force. The horizontal axis represents the total number of channels.

The variation of recognition rates is described for five different classification schemes under different total numbers of channels. All the combinations under the same number of channels were tested and the average recognition accuracy was adopted for subsequent calculations. It is easy to see from Figure 5A that the classification results have a rapid rise at first and then tend to be stable. From Table 2, we can see that at IR5 for SCH4, a negative increase appears for the first time, which represents no improvement in recognition rate with the increase in the total number of channels. For IR7 and IR8, the recognition rates of the five classification schemes are not improved, and the most significant improvement is at IR2, with an average value of 33.34%. These results indicated that the recognition rates of various classification cases tended to be stable at four channels. The performance of subject one under five schemes can be seen in Figure 5B, which is presented in the form of the box plot. Under one channel, the difference between the maximum and the median fluctuates around 10–30%, and the results are unstable. When the total number of channels reaches four, the fluctuation of classification results is 5–10%. Moreover, we can observe that, for the five classification schemes, the increase in the average recognition rate slows down gradually as the number of channels increases. As a result, the total number of four channels was selected to classify the four natural grasping movements.

**TABLE 2:** The increase of rate (IR) when the number of channels increased, compared with the previous number of channels.

Scheme	IR2 (%)	IR3 (%)	IR4 (%)	IR5 (%)	IR6 (%)	IR7 (%)	IR8 (%)
SCH1	15.75	3.90	2.65	0.78	0.21	−0.04	−0.26
SCH2	36.00	5.88	2.36	0.72	0.31	−0.05	−0.15
SCH3	31.02	6.20	2.63	0.78	0.31	−0.15	−0.36
SCH4	31.33	5.65	2.96	−0.50	1.78	0.22	−0.87
SCH5	52.60	7.25	1.10	1.53	1.18	−0.07	−0.18
AVE	33.34	5.78	2.34	0.66	0.76	−0.02	−0.36

**FIGURE 6:**
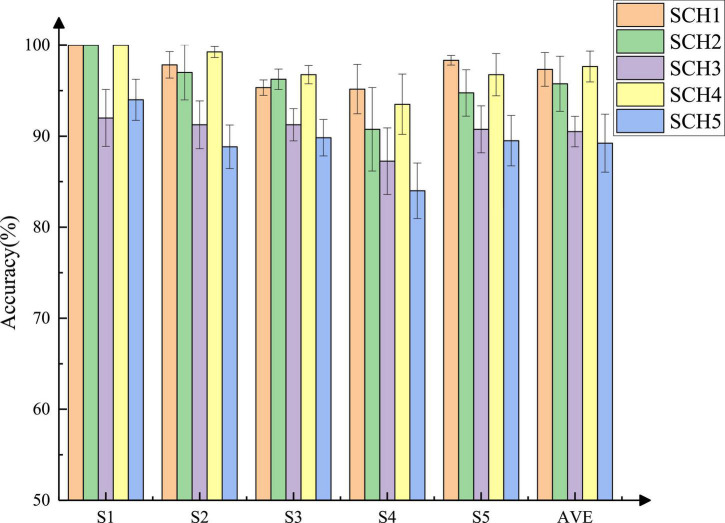
The accuracy of each subject under five classification schemes when the number of channels was four.

Figure 6 shows the classification rates and the averages of five subjects under each classification scheme when the number of channels was four. All the results were verified by the five-fold validation method. The average accuracy of the four-class classification reached 97.33%, the average results of the eight-class classification reached 95.75% (force: level 1 and level 2), 90.5% (force level: level 2 and level 3), and 97.65% (force level: level 1 and level 3), respectively and the average accuracy of the 12 classifications reached 89.23%. Among the eight-class classifications, the results of SCH2 and SCH4 were similar but 5∼7% higher than that of SCH3. Compared with SCH1, the recognition rate of other classification schemes was lower. However, the recognition rate of more than 90% indicated that the classification of four natural grasping movements with force was possible.

### Evaluation of force estimation for natural action

We designed four target force profiles for natural grasping movements, including a rectangular profile, a triangular profile, a step-climbing profile, and a random profile. The step-climbing profile can be regarded as a combination of a rectangular profile and a triangular profile, containing force information from 0 to 80% MVC. In addition, the coefficient of determination (*R*^2^) was chosen for the evaluation of model performance.

**FIGURE 7:**
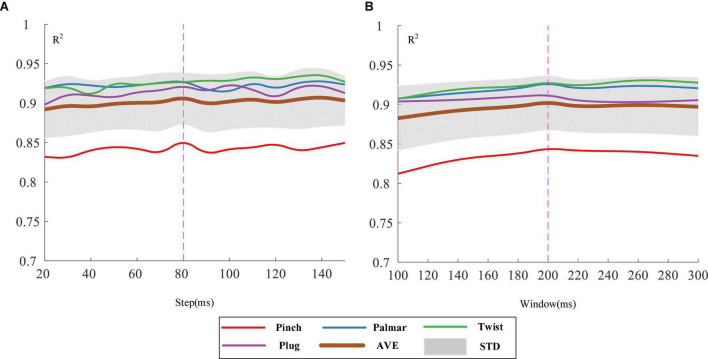
Regression results of the four grasping forces with different step lengths **(A)** and window lengths **(B)**.

**FIGURE 8:**
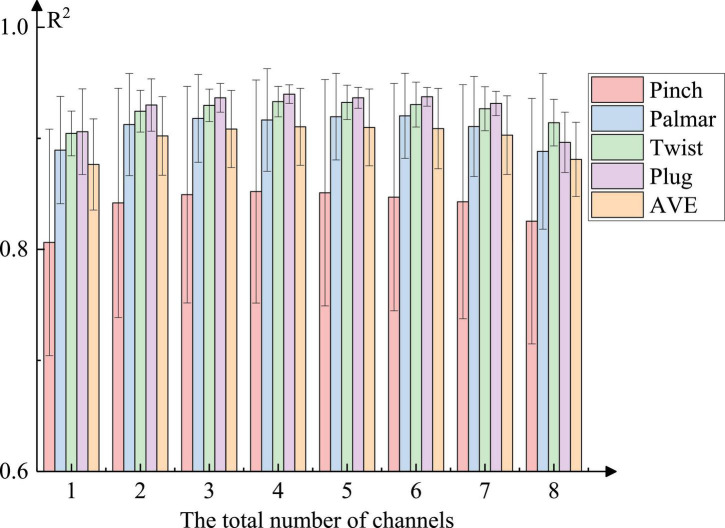
Force estimation results of different natural grasping movements (Take the step-climbing profile as an example). All the combinations were tested and the range of *R*^2^ is indicated. The bars were plotted with the mean values.

1. Selection of sliding windows: We explored the sliding window under the step-climbing profile and eight channels were used simultaneously. The delay between the obtained feedback and the awareness of specific motions that ordinary people can feel is about 300 ms. Therefore, we determined the step of sliding windows under the window size of 300 ms first. The regression performance of different steps could be seen in Figure 7A. The brown curve represents the mean value of the *R*^2^ of four natural grasping movements. Under the window of 300 ms, the step length of 80 ms was the best. Then, with the step size of 80 ms, it was shown in Figure 7B that when the window size reached 200 ms, local optimization was achieved. As result, the sliding window with a window size of 200 ms and a step size of 80 ms were selected for force estimation.

2. Channel selection for force estimation: Figure 8 describes the regression performance of four natural grasping movements regarding the force estimation under different total numbers of channels, which shows an increasing trend followed by a decreasing trend. When the total number of channels reached four, the mean reached the maximum and the regression performance was the best. Additionally, the force estimation of the plug grasp was the best and the standard deviation was the smallest. At the same time, we carried out correlation analysis for eight channels and four natural grasping movements. From Table 3, we could see that correlations were different between these channels and hand movements (*p* < 0.01). It could be also observed that C7 and C8 were not generally related to movements other than plug force. C2, C3, and C4 showed significant strong correlations with all four actions.

**TABLE 3:** Correlation analysis of different channels for grasping movements.

Action types	C1 (%)	C2 (%)	C3 (%)	C4 (%)	C5 (%)	C6 (%)	C7 (%)	C8 (%)
Pinch	81.63 ± 7.44	79.06 ± 4.87	69.83 ± 14.58	69.75 ± 16.95	83.98 ± 3.57	64.92 ± 9.12	10.62 ± 4.09	25.85 ± 17.69
Palmar	73.15 ± 12.87	76.07 ± 11.36	71.97 ± 16.82	80.87 ± 8.86	82.6 ± 5.89	74.13 ± 4.79	27.43 ± 17.95	20.74 ± 22.03
Twist	85.2 ± 3.78	80.07 ± 5.08	80.37 ± 7.63	87.31 ± 3.11	79.92 ± 5.87	79.58 ± 3.61	67.92 ± 13.42	39.44 ± 20.87
Plug	59.26 ± 33.14	77.87 ± 9.66	75.52 ± 11.41	90.01 ± 3.41	86.54 ± 6.34	29.82 ± 18.63	88.92 ± 2.47	49.67 ± 22.14

3. Force estimation for four natural grasping movements: According to Figure 8 and Table 3, we selected different combinations of channels for different natural grasping movements. For pinch grasp, C1, C2, C3, and C5 were selected, for palmar grasp, C2, C4, C5, and C6 were selected, for twist grasp, C1, C2, C3, and C4 were selected, and for plug grasp, C2, C4, C5, and C7 were selected.

**FIGURE 9:**
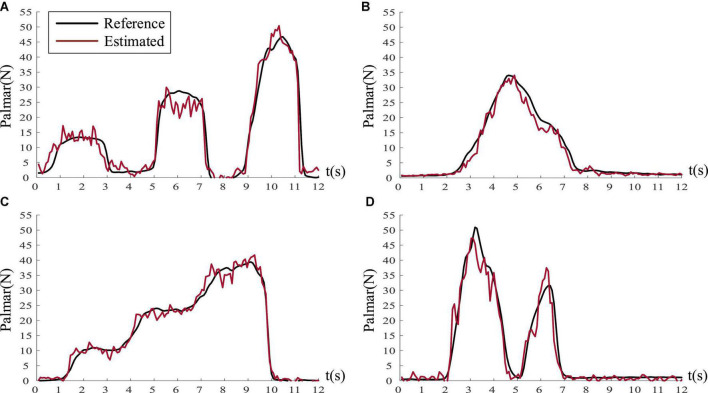
The estimation result of palmar force under four profiles (Take subject five as an example). **(A)** A rectangular profile (*R*^2^ = 0.9375). **(B)** A triangle profile (*R*^2^ = 0.9751). **(C)** A step-climbing profile (*R*^2^ = 0.9832). **(D)** A random profile (*R*^2^ = 0.9485).

**FIGURE 10:**
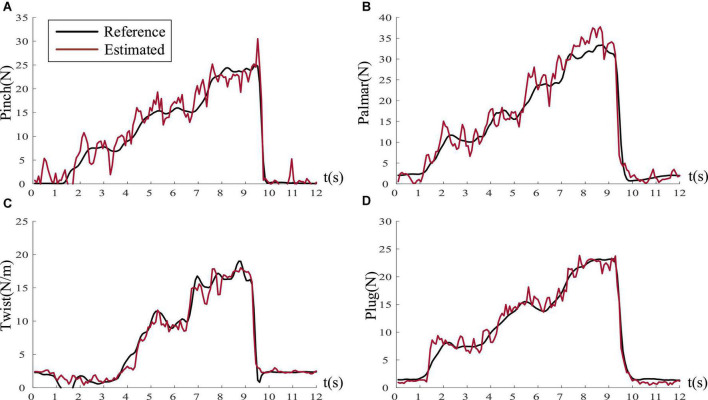
The estimation results of four grasping forces under the step-climbing profile (Take subject three as an example). **(A)** Pinch force (*R*^2^ = 0.9343). **(B)** Palmar force (*R*^2^ = 0.9423). **(C)** Twist force (*R*^2^ = 0.9694). **(D)** Plug force (*R*^2^ = 0.9755).

Figure 9 illustrates the regression results of subject five on palmar force under four task profiles, where the black curve represents the reference force and the red curve is the predicted force. The result shows that good prediction could be achieved by BP network with EMG when subjects performed natural grasping movements. Figure 10 demonstrates the regression results of the four natural grasping movements under the step-climbing profile of subject three. We could observe that the pinch grasp, twist grasp, plug grasp, and palmar grasp achieved almost the same performance. Table 4 shows that it is possible to establish an accurate regression model to predict the force applied by subjects when performing natural grasping movements. Consequently, it is possible to achieve accurate force control of prosthetic limbs or robotic arms.

**TABLE 4:** Coefficient of determination for each individual.

Action types	Force profile	S1 (%)	S2 (%)	S3 (%)	S4 (%)	S5 (%)	AVE (%)
Pinch	Rectangle	79.26 ± 9.06	76.31 ± 7.79	77.22 ± 4.98	79.71 ± 5.83	89.69 ± 4.52	80.44 ± 4.79
	Triangle	87.45 ± 5.86	80.5 ± 11.09	90.53 ± 3.19	91.33 ± 3.44	87.01 ± 6.14	87.36 ± 3.82
	Step-climbing	67.07 ± 8.27	84.84 ± 5.7	89.22 ± 4.39	91.94 ± 3.79	92.5 ± 3.4	85.11 ± 9.42
	Random	81.13 ± 9.72	68.76 ± 9.04	92.1 ± 2.26	89.65 ± 6.88	87.92 ± 6.89	83.91 ± 8.41
	AVE	78.73 ± 7.38	77.60 ± 5.93	87.27 ± 5.89	88.16 ± 4.95	89.28 ± 2.09	84.21 ± 7.45
Palmar	Rectangle	88.52 ± 4.24	74.62 ± 18.75	76.44 ± 5.98	87.59 ± 4.79	89.05 ± 3.29	83.24 ± 6.34
	Triangle	94.56 ± 3.06	82.59 ± 6.85	91.34 ± 3.99	92.97 ± 3.16	92.2 ± 8.65	90.73 ± 4.21
	Step-climbing	91.47 ± 2.5	86.85 ± 6.68	84.07 ± 7.08	85.73 ± 13.48	94.65 ± 1.95	88.55 ± 3.92
	Random	93.3 ± 3.57	74.16 ± 13.26	90.21 ± 8.6	89.16 ± 6.43	90.48 ± 3.48	87.46 ± 6.79
	AVE	91.96 ± 2.27	79.56 ± 5.38	85.52 ± 5.92	88.86 ± 2.66	91.6 ± 2.09	87.5 ± 6.1
Twist	Rectangle	84.74 ± 2.95	81.3 ± 6.61	83.85 ± 6.42	77.33 ± 6.57	77.27 ± 8.06	80.9 ± 3.15
	Triangle	90.68 ± 4.4	92.51 ± 2.65	92.43 ± 3.24	88.02 ± 4.25	81.37 ± 8.64	89 ± 4.15
	Step-climbing	92.2 ± 5.83	91.34 ± 2.67	94.43 ± 1.69	93.46 ± 2.62	87.75 ± 8.19	91.84 ± 2.3
	Random	92.14 ± 3.48	85.07 ± 9.16	93.59 ± 1.88	89.23 ± 5.47	88.95 ± 4.71	89.8 ± 2.94
	AVE	89.94 ± 3.06	87.56 ± 4.59	91.08 ± 4.23	87.01 ± 5.94	83.84 ± 4.76	87.88 ± 5.25
Plug	Rectangle	86.45 ± 2.84	83.59 ± 8.24	85.2 ± 11.31	88.49 ± 3.19	87.32 ± 3.75	86.21 ± 1.7
	Triangle	96.07 ± 2.45	94.81 ± 2.18	94.54 ± 2.5	87.36 ± 11.82	94.5 ± 2.22	93.46 ± 3.1
	Step-climbing	93.17 ± 2.32	91.84 ± 2.68	94.95 ± 2.9	93.77 ± 3.71	90.21 ± 6.96	92.79 ± 1.63
	Random	94.22 ± 2.01	87.27 ± 8.56	93.36 ± 1.88	89.03 ± 6.86	90.24 ± 4.02	90.82 ± 2.61
	AVE	92.48 ± 3.63	89.38 ± 4.29	92.01 ± 3.98	89.66 ± 2.45	90.57 ± 2.56	90.82 ± 3.68

## Discussion

### Classification with force for four natural grasping movements

For a long time, most of the studies about EMG focused on gestures. These researches did not consider the influence of force on the classification results. In this manuscript, for the classification of different movements, a unique experimental apparatus was designed to acquire the force for different grasping movements. We discussed various schemes under different force levels and studied the results under multiple channel combinations. Specifically, we demonstrated the feasibility of identifying different natural grasping movements with force. Meanwhile, with the increasing number of channels, the recognition rate of each classification scheme also improved significantly. When the total number of channels reached four, we observed that the classification accuracy would not improve.

Cracchiolo et al. (2021) designed three different levels of grip force to classify different forces and achieved a recognition rate of 72.2%. Khan et al. (2021) also classified different levels of pinch grasping forces, with an accuracy ranging from 91.7 to 94.5%. However, their work was to investigate the possibility of identifying different levels of force in one motion. It is difficult to directly compare our study with theirs. Our work demonstrates achieving natural force control for prostheses and robot arms is possible.

### Performance of regression

For the force estimation, the effect of different sliding windows on the regression was investigated, and the window length of 200 ms and the step length of 80 ms were established as the best. Then, to compare different channel combinations, we analyzed the correlation between the EMG signals and natural grasping movements. Finally, the best channel combination under different movements was established and the regression models were built based on the selected channels. In our study, we found the feasibility of classification for different actions with force, which indicated that EMG was closely related to the force information. Furthermore, literature (Martinez et al., 2020b) also showed that force estimation could be performed by EMG. Meantime, most researchers mainly focused on the regression of palmar force or pinch force and did not explore the force estimation of other natural hand motions. In this research, we investigated force estimation of four natural grasping movements: pinch, palmar, twist, and plug grasp.

1. Selection of the best channel: We performed force estimation for four movements with different channel combinations and found that the best regression was achieved when the total number of channels was four, but the optimal channel combination was not the same for each movement. According to the correlation analysis in Table 3, we chose different combinations for these movements. For the pinch grasp and twist grasp, flexor carpi radialis, extensor digitorum, and palmaris longus were involved more. For the palmar grasp and plug grasp, extensor digitorum, brachioradialis, and extensor carpi radialis were used more. This suggests that we need to consider the relationship between different muscle groups when using EMG for action decoding and force estimation.
  In the research of Castellini et al. (2008), electrodes were similarly placed on the hand and forearm to acquire sEMG signals and features were extracted subsequently. However, the channels were not optimized, which might lead to redundant information and less accuracy. Hoozemans and van Dieen (2005) used 6 forearm muscles to predict the palmar force and found that using 3, 4, or 5 muscles to predict force was as effective as using all muscles, which was in accord with our study. He speculated that the random samples of three muscles could produce enough validity for model prediction and the positions of some electrodes in the forearm were irrelevant. Regarding correlation, we also found that not all arm muscles were closely related to grasping movements.
2. Force estimation of different natural grasping movements: We conducted force estimation experiments for four natural movements on five subjects. Regarding palmar grasp, the mean *R*^2^ is 0.9465 under the step-climbing profile. Compared with palmar grasp, the regression of twist grasp and plug grasp gets better performance. Pinch grasp has relatively lower regression performance, probably due to the small amplitude of the movement and the fewer muscle groups called. The regression of plug grasp can reach 0.9607 under the triangular profile, and the average *R*^2^ under the four profiles can attain more than 0.8938.
  Different performance for each action is observable. Kundu et al. (2014) gave different stimulation to large nerve animals, and the muscle mass responded differently. This showed that for different natural grasping movements, the muscle potentials of the eight muscles would be very different. We speculate that the poor performance of pinch grasp may be related to the applied force, which is smaller than the other three movements and may call for a smaller number of muscle groups as well as a smaller degree of muscle response. Zhang et al. (2021) confirmed that the muscle coordination of palmar grasp and pinch grasp was different under different force levels. Unfortunately, there is little research in this area for us to compare force estimation of different grasping movements. This suggests that researchers should not simply rely on machine learning, but need to focus on the connection between muscles and motions from a physiological perspective.
3. Force estimation under different task profiles: For each natural grasping movement, we designed four task profiles that covered the magnitude of the subject’s force from 0 to 80% MVC, which contained rich force information. We found that subjects performed best under the triangular task profile, followed by the step-climbing profile and the random profile, and the rectangular profile was the worst. It is believed that the reason may be how the subjects applied the force. Subjects applied and released force slowly in the triangular profile task, and there were jumps in force application under the other tasks.
  Among numerous research, experimental tasks vary from study to study. In Hu’s study, eleven gestures including pressure, pinch, grip, and twist were extracted from the typical gestures in daily life. They designed three types of gesture acquisition tasks: (1) the Maximal Voluntary Contraction Task; (2) the Regular Force Pattern Task; (3) the Self-Selected Force Pattern Task. The Regular Force Pattern Task contained two parts, one of which was the sinusoidal force curve and the other was the constant force curve. The former was set to collect data for classification and regression models while the latter was conducted to obtain data for the training of their proposed algorithm based on the threshold method (Hu et al., 2022). Zhang et al. (2018) adopted linear and constant force profiles when estimating the muscle force. However, the reasons for the choices of force curves were not clearly mentioned in such articles and it is necessary to investigate the impact of different profile tasks. These will bring new insights to our next research: To study the influence of different force application methods on the force estimation of EMG. The work of Mamidanna et al. (2021) on the grasping force of EMG prostheses also showed that better force estimation could be achieved by sacrificing the speed of grasping movement. Bog et al. (2011) also designed different task profiles for grasping when studying the influence of different EMG characteristics on the estimation of palmar grasp, and found that the slope of the profile might lead to changes in the regression effect. The findings of Mamidanna and Bog are similar to our research, and the speed of applying force can be clearly reflected in the slope of the profile. Under the triangular profile and step-climbing profile, the force application speed of subjects is slow, and the performance of regression is the best. In contrast, the force application speed of arbitrary profiles and rectangular profiles is faster, and the performance is lower. In future work, we will make a more detailed experiment to investigate the effect of force speed.

### Limitations

Our work is based on offline analysis, and online experiments will put forward higher requirements for real-time performance and accuracy of the algorithm. EMG-based robotic arm control is the future work we envision, but it is a complex project that involves techniques such as path planning. Secondly, our work is based on healthy people and needs to consider more conditions for amputees. The terminal muscle nerves and incomplete muscle groups of amputees or limb deformities are our new challenges. A richer group of subjects will provide more data sets for better evaluation.

## Conclusion

In this manuscript, we conducted a classification and force estimation study for four natural grasping movements. On thek one hand, we presented the high possibility of the classification for these actions with different levels of force. On the other hand, we investigated the feasibility of force estimation for natural grasping movements and found that the regression performance of plug grasp was the best among the four natural grasping movements. Furthermore, muscles responded differently to different actions. Finally, we found that the speed of force application affects the regression results of EMG. Our work is of great significance to the force control of myoelectric prostheses and EMG-based rehabilitation training systems.

## Data availability statement

The raw data supporting the conclusions of this article will be made available by the authors, without undue reservation.

## Ethics statement

The studies involving human participants were reviewed and approved by Ethics Committee of Southeast University. The patients/participants provided their written informed consent to participate in this study.

## Author contributions

BX and KZ designed the study, analyzed the data, and wrote the manuscript. KZ and DL set up the experiment platform. XY performed the experiment. CH, HL, and AS were involved in the critical revision of the manuscript. All the authors read and approved the final manuscript.
